# Effect of PFOA exposure on diminished ovarian reserve and its metabolism

**DOI:** 10.1186/s12958-023-01056-y

**Published:** 2023-02-01

**Authors:** Haofei Shen, Min Gao, Qiuyuan Li, Huipeng Sun, Yingdi Jiang, Lihong Liu, Jingyuan Wu, Xiao Yu, Tianyu Jia, Yongan Xin, Shiqiang Han, Yiqing Wang, Xuehong Zhang

**Affiliations:** 1 Lanzhou University, Lanzhou, 730000 Gansu China; 2 Lanzhou University First Affiliated Hospital, Lanzhou, 730030 Gansu China; 3 Gansu International Scientific and Technological Cooperation Base of Reproductive Medicine Transformation Application, Lanzhou, 730030 Gansu China; 4 Linxia Hui Autonomous Prefecture Maternity and Childcare Hospital, Linxia, China

**Keywords:** Perfluorooctanoic acid (PFOA), Diminished ovarian reserve, Follicular fluid, In vitro fertilization, Embryo quality, Metabolite

## Abstract

Owing to its difficulty in degrading and ease of accumulation in the body, perfluorooctanoic acid (PFOA) has a detrimental effect on reproduction. This study aimed to examine the effect of PFOA concentration in follicular fluid during ovulation stimulation on embryo quality and the impact of PFOA exposure on the metabolic components of follicular fluid. This was a single-center prospective study that included 25 patients with diminished ovarian reserve (DOR), 25 with normal ovarian reserve (NOR), and 25 with polycystic ovary syndrome (PCOS). Follicular fluid samples were analyzed using ultra-high performance liquid chromatography-tandem mass spectrometry. We demonstrated that the PFOA levels of follicular fluid in the DOR group were higher than those in the NOR group and PCOS group (*P* < 0.05). PFOA concentration in the PCOS group was negatively correlated with high-quality embryos (*P* < 0.05). To gain more insight into the impact of PFOA on the metabolic composition of follicular fluid, we classified the DOR group based on the PFOA concentration, for which metabolomic analysis was performed. In the high-concentration PFOA group, there was an increase and a decrease in three and nine metabolites, respectively, compared to that in the low-concentration group. These results suggest that PFOA may alter the metabolic composition of follicular fluid, thus, affecting ovarian reserve function.

**Supplementary Information:**

The online version contains supplementary material available at 10.1186/s12958-023-01056-y.

## Introduction

Endocrine-disrupting compounds (EDCs) are a group of chemicals exhibiting various cytotoxic effects via different mechanisms, thus, interfering with the normal physiological activities of cells and tissues [1]. Substantial evidence confirms the adverse effects of EDCs on female reproductive health [2]. Per- and polyfluoroalkyl substances (PFAS) are man-made organic compounds comprising fluorine atoms and specific functional groups attached to the backbone of a carbon chain [3]. Owing to the molecular stability of PFAS, it is resistant to water, oil, heat, and surface activity [4]. PFAS are currently used in kitchenware, food packaging, cleaning products, and fireproofing foam [5]. PFAS can enter the body through various routes, including inhalation, ingestion, and cutaneous contact [6]. It has been reported that food consumption and PFAS concentration are positively correlated, making food the primary route through which humans are exposed to PFAS [7, 8]. PFAS can be detected in the blood, urine, follicular fluid, and breast milk of an individual [9–11]. PFAS is difficult to degrade and has a half-life of 4 − 5 years [12]. PFAS can disrupt the endocrine system, thus, leading to reproductive health problems such as female infertility or hormonal imbalance [11].

Research has identified over 5,000 chemical structures of PFAS, of which perfluorooctanoic acid (PFOA) is the most exposed and widely studied representative compound [13]. The end-metabolite of PFAS, known as PFOA, is also employed in processing aid and fire-fighting foams [14]. Both epidemiological and in vitro research suggest that PFOA substances harm reproductive health. Studies have used animal or in vitro cellular experiments to demonstrate that PFAS may affect fertility by impairing the synthesis of sex hormones and their receptors. A study has observed a potential link between PFOA and the development of polycystic ovary syndrome (PCOS) [15]. Women with endometriosis exhibit higher levels of PFOA [16]. Studies have reported that PFOA exposure is associated with delayed menarche and irregular menstrual cycles [17]. Exposure of 17 days post-coitum mouse CD-1 to high levels of PFOA can induce apoptosis in oocytes [18]. High plasma concentrations of PFOA can cause a decrease in the 2 pro-nuclei rate during in vitro fertilization (IVF) [1].

Most studies have focused on serum and urine PFOA levels, and few studies have focused on changes in PFOA in follicular fluid. Follicular development is strictly regulated by intra- and extra-ovarian factors, including follicular fluid, oocyte, and granulosa cell composition, and any link that is affected can cause aberrant oocyte development [19]. The microenvironment of follicular fluid is important for oocyte development. PFOA may cross the blood barrier and be exposed during development. Few studies have examined the relationship between PFOA levels in follicular fluidand embryo quality in IVF. Therefore, our study aimed to determine whether associations exist between the following: (1) PFOA concentration in follicular fluidin the PCOS group and diminished ovarian reserve (DOR) group. (2) PFOA concentration in follicular fluidand embryo quality. Further, we explored the metabolic components of follicular fluid that are altered by high PFOA exposure. This study sought to determine the PFOA exposure in various populations and the effects of PFOA on the metabolite composition in the follicular fluid to investigate their potential effects on oocytes.

## Materials and methods

### Patients and sample collection

Our study recruited 75 females undergoing IVF-embryo transfer treatment between January 2022 and August 2022 in Lanzhou University First Affiliated Hospital. There were 25 females in the normal ovarian reserve (NOR) group, 25 in the PCOS group, and 25 in the DOR group based on the inclusion and exclusion criteria. The study was approved by the Clinical Research and Ethics Committee of the Lanzhou University Affiliated First Hospital, and informed written consent was obtained from all patients (Ethics Number: LDYYSZLL2022-02). The diagnosis of DOR was made according to the Bologna criteria [20]. The diagnosis of PCOS was based on the Rotterdam ESHRE/ASRM-sponsored PCOS consensus [21]. Exclusion criteria for both groups included the following: History of laparoscopic surgery and pelvic surgery; patients with ages > 40 years;chromosomal abnormalities; antibiotic treatment within 3 months. The progestin-primed ovarian stimulation technique was used to stimulate the ovaries of all recruited females. The data that were accessible included age, body mass index (BMI), infertility type, menarche age, gravidity, parity, basal blood hormone, number of retrieved oocytes, number of transferred embryos, and embryo quality. Follicular fluid was collected during oocyte retrieval and contaminated follicular fluid with blood was not included in this study. Follicular fluid collected in the test tube was centrifuged at 3000 rpm for 10 min, and the supernatant was collected freezing preservation in a refrigerator at -80 ℃. High-quality embryo rate = Number of (Grade 1embryo + Grade 1embryo)/Total number of retrieved embryos × 100%. High-quality embryo = Grade 1 + Grade 2. Grade 1 embryos had 6–10 cells, a fragmentation of 0–10%, and perfect symmetry; Grade 2 embryos had either 6–10 cells with fragmentation of 0–25% and perfect or moderate symmetry or they had 4–6 or > 10 cells with fragmentation of 0–10% and perfect symmetry [22].

### Sample preparation

The fluid was thawed at room temperature, and 0.5 mL fluid was collected into a 15-mL centrifuge tube. Two milliliters of Na_2_CO3 buffer solution (0.25 mol/L), 5 mL tert-butyl methyl ether (MTBE), and 1 mL tetrabutylammonium hydrogen sulfate (0.25 mol/L) were successively added into the centrifuge tube. After capping, it was placed on the thirsty rotor for 2–3 min. The tube was placed in the ultrasonic extraction instrument for 5 min and then centrifuged at 2500 rpm for 10 min. The upper MTBE was transferred to a new 15-mL tube once the liquid had stratified. These steps were repeated thrice before extracting the liquid. The solution was heated to 45 ℃, dried with high-quality hydrogen, and dissolved in 1 mL of methanol. The solution was filtered by 0.22-mm nylon and then placed in a sample vial for chromatography.

### Ultra-high performance liquid chromatography-tandem mass spectrometry analysis (UHPLC-MS/MS)

Target PFAS were analyzed using an Agilent Technologies UHPLC-MS/MS, comprised of a 1290 Infinity II high-speed pump (model G7120A) connected to a triple quadrupole (model G6470C) mass spectrometer and Jet Stream ESI source (Agilent Technologies Inc., Santa Clara, CA, USA). The UHPLC pump has a switching valve that minimizes impurities from repeated sample analysis, increases column life, and minimizes ion source contamination. Chromatographic separation was done using a Rapid Resolution High Definition (RRHD) Eclipse Plus C18 column (2.1 × 100 mm, 1.8 μm; Agilent Technologies, USA) connected to an RRHD Eclipse Plus C18 pre-column (2.1 × 50 mm, 1.8 μm; Agilent Technologies, USA). A gradient elution procedure was used for the liquid phase separation of the target. Mobile phase A was 5 mM ammonium acetate; mobile phase B was chromatographic grade methanol. The program was as follows: 0 − 1 min, 10 − 40% B; 1 − 4 min, 40 − 95% B; 4 − 4.1 min, 95 − 10% B; 4.1 − 5 min, 10% B. The flow rate was 0.3 mL/min; the column temperature was 30 °C; the injection volume was 5 μL.

The qualification and quantitative determination of PFASs were performed under negative heated ESI (HESI) and operated in parallel reaction monitoring mode. The conditions of the mass spectrometer were as follows: The HESI spray voltage was ± 3.5 kV; the S-lens RF level was 50. The capillary temperature and aux gas heater temperature were 350 °C and 450 °C, respectively. The sheath gas flow rate, aux gas flow rate, and sweep gas flow rate were 25, 5, and 0 arb units, respectively. The MS resolution and targeted MS2 resolution were 70,000 and 35,000. The automatic gain control target value was 5 × 10^4^, and the maximum injection time adopted an automatic value. Data were processed using the Xcalibur software.

### Metabolomics of follicular fluid

The DOR group was classified into high- and low-concentration groups based on the concentration of PFOA exposure and subjected to metabolomic sequencing. High-concentration groups showed PFOA $\ge$ 38 ng/ml and low-concentration groups showed PFOA $<$ 38 ng/ml. The follicular fluid was collected in a test tube and centrifuged at 3000 rpm for 10 min, and the supernatant was collected for frozen preservation at -80 ℃. The follicular fluid samples were thawed before 200 μL was applied to the extraction procedure; the samples were mixed with 800 μL methanol: acetonitrile (4:1, v/v) solution and an internal standard of 0.02 mg/mL L-2 chlorophenylalanine. The mixture was allowed to settle at -10 ℃ and treated with a high-throughput tissue crusher Wonbio-96c (Shanghai Wanbo Biotechnology Co., Ltd.) at 50 Hz for 6 min, followed by ultrasound examination at 40 kHz for 30 min at 5 ℃. The samples were placed at -20 ℃ for 30 min to precipitate the proteins. Following centrifugation at 13,000 × g at 4 ℃ for 15 min; the supernatant was carefully transferred to sample vials for LC–MS/MS analyses. A polled quality control sample (QC) was established by combining similar quantities of all samples as a part of the system conditioning and QC procedure. Procedures similar to those applied for sample analysis were used to examine and dispose of the QC samples. It was used to represent the whole sample set, which was injected at regular intervals to monitor the stability of the analysis.

The instrument platform used for LC–MS analysis was the UHPLC-Q Exactive HF-X system (Thermo Fisher Scientific) under the following chromatographic conditions: 2 μL of the sample was separated on an HSS T3 column (100 × 2.1 mm i.e., 1.8 μm) and further detected using mass spectrometry. The mobile phases comprised 0.1% formic acid in water:acetonitrile (95%:5%, v/v) (solvent A) and 0.1% formic acid in acetonitrile:isopropanol:water (47.5%:47.5%:5%, v/v) (solvent B). The following rates caused a change in the solvent gradient and were used for equilibrating the systems: 0–3.5 min, 0–24.5% B (0.4 mL/min); 3.5–5 min, 24.5–65% B (0.4 mL/min); 5–5.5 min, 65–100% B (0.4 mL/min); 5.5–7.4 min, 100% B (0.4–0.6 mL/min); 7.4–7.6 min, 100–51.5% B (0.6 mL/min); 7.6–7.8 min, 51.5–0% B (0.6–0.5 mL/min); 7.8–9 min, 0% B (0.5–0.4 mL/min); 9–10 min, 0% B (0.4 mL/min). The sample injection volume was 2 µL, and the flow rate was set to 0.4 mL/min. The column temperature was maintained at 40 ℃. During the analysis, all these samples were stored at 4 ℃ under MS conditions. The mass spectrometric data were collected using the Thermo UHPLC-Q Exactive HF-X Mass Spectrometer equipped with an ESI source operating in either positive or negative ion mode. The optimal conditions were set as follows: Heater temperature, 425 ℃; Capillary temperature, 325 ℃; sheath gas flow rate, 50 arb; aux gas flow rate, 13 arb; ion-spray voltage floating, -3500 V in the negative mode and 3500 V in the positive mode, respectively; normalized collision energy, 20 − 40 − 60 V rolling for MS/MS. Full MS resolution was 60,000, and MS/MS resolution was 7500. Data acquisition was performed in the Data Dependent Acquisition mode. The detection was conducted over a mass range of 70–1050 m/z.

Following data acquisition, the peaks were first filtered for a ery low signal undetectable, b. detection errors, such as ion suppression or instrument performance instability, and c. algorithmic limitations of peak extraction. Further, the peaks were identified, and the peak areas were calculated. Subsequently, the missing data were filled using the minimum value. Finally, normalization was performed using the median. All these steps were performed using Major BIOS software.

### Differential metabolites analysis

Partial least squares discriminant analysis (PLS-DA) and orthogonal PLS-DA (OPLS-DA) models of high-and low-concentration groups were constructed using R package ropls, while the determination of variable importance in projection (VIP) values of metabolites in each model were calculated. Subsequently, the metabolites with significant differences in high-and low-concentration groups were identified using *P* < 0.05, difference multiples > 1, and VIP value > 1 as the screening criteria, and the screening process was demonstrated by volcano plots. Further, the top 12 metabolites with the most significant differences between the groups were screened. All these steps were performed using Major BIOS software.

### Statistical analysis

The statistical analyses were performed using IBM SPSS 22.0 and empower stats based on the R language. Continuous variables were presented as means ± standard deviation, and a t-test was used for comparisons. If normality was not satisfied, the Mann − Whitney U test was used for comparisons. Categorical variables were presented as a percentage. *P* < 0.05 was considered statistically significant. In this study, PFOA exposure concentrations in the DOR group were grouped by quartile method, Q1 (4.45 − 13.96 ng/mL), Q2 (14.29 − 38.67 ng/mL), Q3 (62.86 − 155.82 ng/mL), and Q4 (182.23 − 485.43 ng/mL). Single-factor logistic regression analysis based on a generalized estimating equation was used to study the effect of PFOA in follicular fluid on the rate of high-quality embryos. A curve-fitting analysis based on generalized additive mixed modeling (GAMM) was used to investigate the relationship between PFOA in follicular fluid and the rate of the high-quality embryo.

## Results

### Clinical characteristics

This study included 75 individuals without antibiotic usage and compared 25 females in the NOR group, 25 females in the DOR group, and 25 females in the PCOS group. The baseline features of patients are presented in (Table 1). There were no significant differences in BMI, gravidity, parity, primary infertility rate, and fertilization method between the three groups (*P* > 0.05). The anti-Müllerian hormone and the number of retrieved oocytes were significantly higher in the PCOS group than those in the NOR and DOR groups (*P* < 0.05). Details of the PFOA-related mass parameters are displayed in (Table 2).

**Table 1:** Comparison of clinical characteristics among group DOR, NOR and PCOS

Variables	DOR	NOR	PCOS	DOR vs. NOR *p*-value	PCOS vs. NOR *p*-value
Total NO. of patients	25	25	25		
Age(year)	34.00 ± 3.07	32.08 ± 3.71	29.84 ± 3.93	0.05	0.04
Body mass index(kg/m^2^)	23.54 ± 4.00	22.82 ± 2.73	23.74 ± 3.95	0.46	0.35
Gravidity	0.96 ± 1.17	0.84 ± 1.14	0.31 ± 0.63	0.72	0.10
Parity	0.36 ± 0.57	0.28 ± 0.42	0.08 ± 0.27	0.30	0.51
Menstrual cycle	30.04 ± 7.14	30.40 ± 7.88	45.28 ± 22.53	0.77	0.00
Primary infertility rate(%)	(12) 48	(13) 52	(18) 72	0.78	0.15
Second infertility rate (%)	(13) 52	(12) 48	(7) 28		
FSH	9.66 ± 5.20	7.84 ± 2.57	5.76 ± 1.01	0.14	0.00
LH	5.05 ± 2.75	6.10 ± 3.97	8.93 ± 4.79	0.35	0.01
E2	49.59 ± 50.95	36.79 ± 14.37	35.48 ± 18.99	0.83	0.49
AMH	0.57 ± 0.39	1.9 ± 0.57	8.8 ± 4.58	0.00	0.00
IVF(%)	(18) 72	(14) 56	(15) 60	0.38	0.77
ICSI(%)	(7) 28	(11) 44	(10) 4		
Number of retrieved oocytes	3.48 ± 1.26	9.28 ± 3.92	19.40 ± 6.61	0.00	0.00

*NOR* Normal ovary response, *DOR* Diminished ovarian reserve, *PCOS* Polycystic ovarian syndrome, *FSH* Follicle-stimulating hormone, *LH* Luteinizing hormone, *E2* 17- estradiol, *AMH* Anti-müllerian hormone, *IVF* In vitro fertilization, *ICSI* Intracytoplasmic sperm insemination

**Table 2:** The HESI-MS related parameters for PFOA

Analyte	Abbreviation	Chemical Formula	Molecular weight	Parent ion (m/z)	Product ion (m/z)	NCE (eV)	RT (time)
Perfluorooctanoic acid	PFOA	C8HF15O2	414.07	412.96	368.97	10	4.17

### PFOA concentration in follicular fluid

**Fig. 1:**
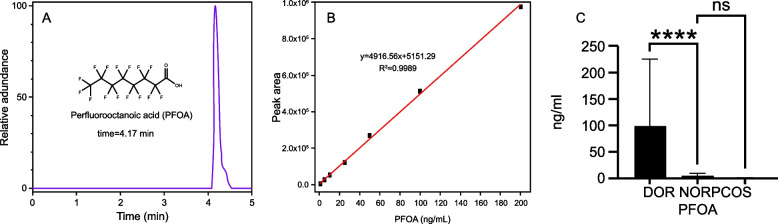
**A** PFOA chemical formula. **B** PFOA standard curve. **C** Distribution of PFOA in different groups

PFOA molecular formula and standard curve are depicted in (Fig. 1 A-B). The concentration of PFOA in DOR group was considerably higher than that in the NOR and PCOS groups (Fig. 1 C).

### The association between PFOA and embryo quality

**Fig. 2:**
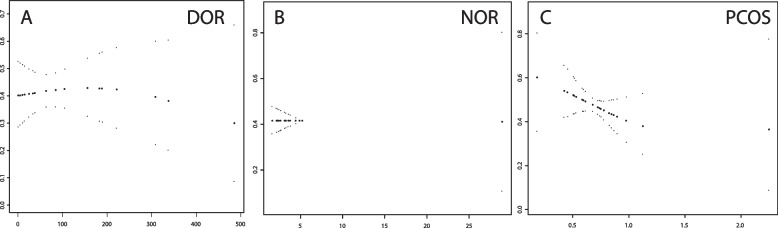
GAMM model of the relationship between PFOA concentration in follicular fluid and high-quality embryo rate. The abscissa indicates the PFOA concentration (ng/ml) in follicular fluid, the ordinate indicates the good-quality embryo during IVF. **A** DOR group. **B** NOR group. **C** PCOS group

GAMM curve analysis was used to study the relationship between the level of PFOA in follicular fluid and the high-quality embryo rate in the population. PFOA concentration in the PCOS group was negatively correlated with high-quality embryos (*P* < 0.05). The DOR and NOR groups did not exhibit a significant difference in the POFA concentration (*P* > 0.05) (Table 2) (Fig. 2 A-C).

### Overall metabolic profile and QC of untargeted metabolomics analysis

**Fig. 3:**
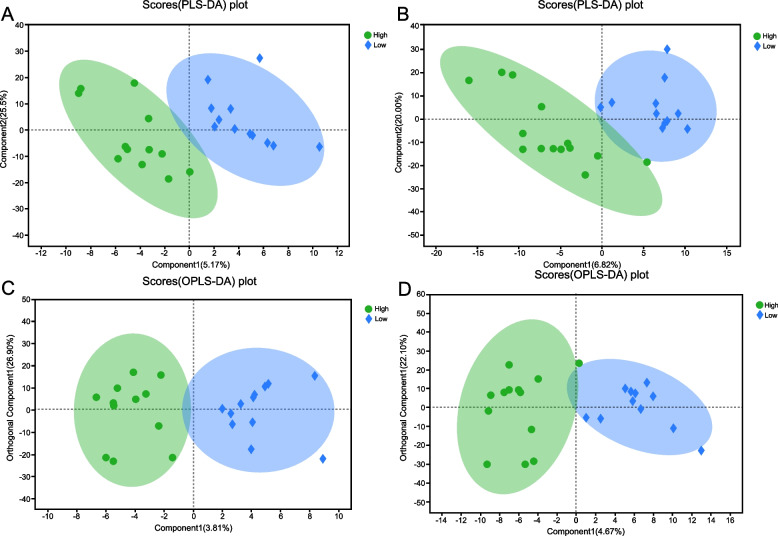
Partial least squares discriminant analysis (PLS-DA) score from follicle fluid metabolomics profiles comparing high concentration group and low concentration group (A-B). **A** Positive ESI mode; **B** Negative ESI mode. Orthogonal partial least squares discriminant analysis (OPLS-DA) score from follicle fluid metabolomics profiles comparing high concentration group and low concentration group (C-D). **C** Positive ESI mode. **D** Negative ESI mode

A total of 140 metabolites were detected using the LC–MS/MS method, which included 100 metabolites in the negative ESI (ESI-) mode and 40 metabolites in the positive ESI (ESI +) mode. To assess the amount of variance between the high- and low-concentration groups, the FF metabolomic profiles were thoroughly compared using PLS-DA (Fig. 3. A-B) and OPLS-DA (Fig. 3 C-D). The R2Y(cum) of OPLS-DA performed to compare the metabolites between high- and low-concentration groups in the ESI + and ESI- modes were 0.853 and 0.841, respectively. (Fig. 3 C, D).

### Metabolites in high- and low-concentration groups

**Fig. 4:**
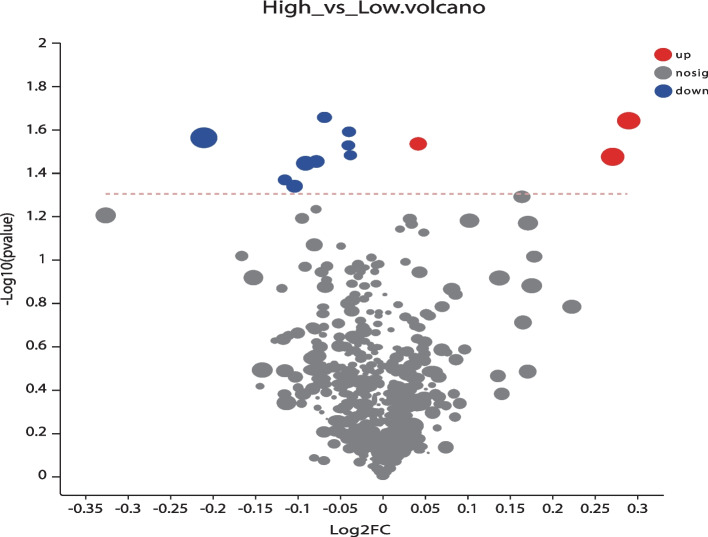
Volcano plot of metabolite components based on the normalized peak intensity between high concentration group and low concentration group. Colored dots indicate metabolites with a VIP value greater than 1, while the gray dots indicate the remaining metabolites. Red for up-regulated and blue for down regulated. The closer to the left and right side and the top point, the more significant the difference

**Fig. 5:**
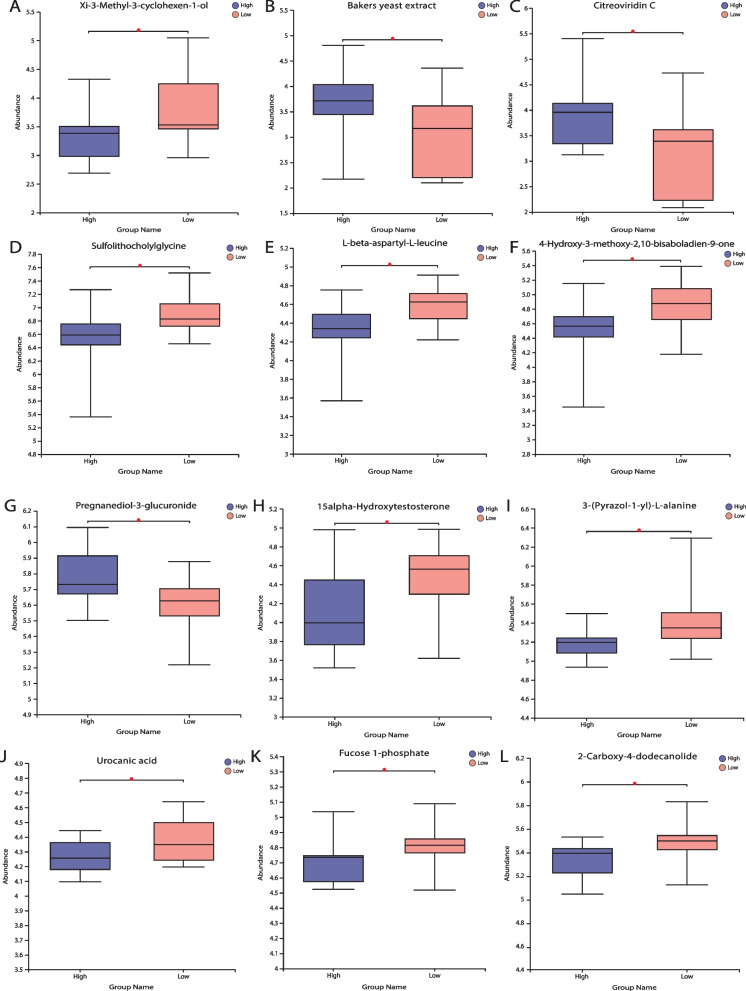
Significance of metabolites in between groups

The chemical structures of the metabolites were identified using an online database. For the univariate analysis, specific biomarkers were compared among the DOR, NOR, and PCOS groups using the Student’s t-tests. Thus, the metabolite compounds with VIP > 1 and *P* < 0.05 were considered significant (Fig. 4). In the high-PFOA concentration group, three metabolites were increased and nine were decreased compared to those in the low-concentration group. Metabolites with significantly elevated expressions include baker’s yeast extract, Citreoviridin C, and pregnanediol-3-glucuronide. Conversely, metabolites with significantly decreased expression were xi-3-methyl-3-cyclohexen-1-ol, sulfolithocholylglycine, L-beta-aspartyl-L-leucine, 4-hydroxy-3-methoxy-2, 15alpha-hydroxytestosterone (15alpha-T), 3-(pyrazol-1-yl)-L-alanine, urocanic acid (UCA), Fucose-1-phosphate, and 2-carboxy-4-dodecanolide (Fig. 5 A-L).

## Discussion

In this study, we observed the presence of PFOA in the follicular microenvironment. We also reported that PFOA exposure varies in different populations. PFOA exposure potentially affected embryo development, especially in the PCOS population during IVF (Table 3). China is the primary producer and largest consumer of PFAS [23]. PFOA and PFOS, the most commonly exposed PFAS, have been confirmed to have toxic effects on numerous human systems. However, owing to its widespread use in consumer goods and long half-life, it is difficult to be degraded in vivo [24]. This study aimed to explore the effects of PFOA on the female reproductive system through clinical studies and provided a theoretical basis for further research.

**Table 3:** Relationship between PFOA level and embryo quality rate in follicular fluid

PFOA DOR (ng/ml)	Q1 (4.45–13.96)	Q2 (14.29–38.67)	Q3 (62.86–155.82)	Q4 (182.23–485.43)	*P* value
	35.71% (2/12)	41.67% (5/12)	53.33% (8/15)	33.33% (5/15)	0.471
PFOA NOR (ng/ml)	Q1 (1.69–2.67)	Q2 (2.68–3.32)	Q3 (3.54–3.64)	Q4 (3.75–28.89)	
	30.95% (13/42)	47.50% (19/40)	55% (22/40)	39.22% (20/51)	0.140
PFOA PCOS (ng/ml)	Q1 (0.19–0.53)	Q2 (0.59–0.68)	Q3 (0.73–0.78)	Q4 (0.83–2.25)	
	51.28% (40/78)	47.31% (44/93)	60.76% (48/79)	32.65% (32/98)	0.002

Considering that PFAS can cross the blood-follicle barrier and persist in follicular fluid, PFOA has a greater chance of coming in contact with and damaging oocytes directly [9]. Previous studies have reported that PFOA levels are elevated in the follicular fluid of females with infertility, indicating that PFOA is linked to poor fertility [11, 25]. Recently, animal experiments have indicated that the impact of PFOA on oocyte maturation and embryo development should be evaluated. It has been reported that exposure to PFOA during prepuberty caused a decline in the number of follicles and more frequent irregular estrous cycles [26]. PFOA exposure also altered follicle count, manifested as decreased primordial follicles and increased preantral and antral follicles, in adult mice. Additionally, estradiol and estrone levels also decreased under the influence of PFOA [27]. Certain evidence has verified that PFOA can induce metabolic disorders by activating the peroxisome proliferator-activated receptor family (PPARs) which are considered to be involved in gametogenesis and embryo development [28]. For example, the expression of PPARγ is regulated by the luteinizing hormone and influences the production of estradiol and progesterone in granulosa cells [29]. Thus, the combination of PFOA and PPARs may interrupt steroid hormone production, thus, adversely affecting oocyte and embryo. Huang et. al observed that PFOA exposure in vivo impaired oocyte meiosis and preimplantation development by oxidative stress. PFOA decreased the size of antral follicles in vitro and reduced the ovarian response to superovulation [30]. The impact of PFOA on oocytes and embryos can be an explanation for poor fertility and adverse IVF outcome.

In this study, we analyzed the relationship between high and low concentrations of PFOA in follicular fluid during ovulation stimulation and embryo quality. The population is grouped in this study, which is novel as it has not been done before. Previous studies have demonstrated that higher serum levels of PFAs are associated with high-quality embryo rates [31]. A single-cell transcriptome analysis found that PFOA induces oocyte deterioration by affecting mitochondrial dysfunction and apoptosis in the offspring [32].Significantly lower fertilization rates have been detected in PFAS follicular fluid [33]. Studies have reported a positive correlation between PFOA serum concentration and PFOA expression in follicular fluid. A study has observed that PFOA in serum was negatively associated with the number of retrieved oocytes, mature oocytes, and good-quality embryos[1]. PFOA exposure was observed to be associated with PCOS, however, our findings revealed low expression levels of PFOA in both PCOS and NOR groups. Population variations were observed while comparing the relationship between PFOA and embryo quality in this study. The DOR group had the higher PFOA exposure, however, the GAMM model analysis indicated no effect on the embryo fate. Notably, we identified PFOA to be a risk factor for DOR, and the discrepancy in results could be attributed to the small sample size.

To further understand the impact of PFOA on metabolites in follicular fluid, we performed a non-targeted metabolomic analysis of the DOR group based on the high- and low-PFOA concentrations and observed significant differences in 15alpha-T and UCA expression. A study suggests that estrogen receptor activation may exert immunoprotective effects by scavenging cis-UCA-induced reactive oxygen species and preventing cis-UCA immunosuppression [34]. Young et al. revealed that 15α-T can respond to estrogen and progesterone in a way that is similar to different forms of gonadotropin-releasing hormones, more than even classical sex hormones, with a dose–response relationship [35]. All these metabolite changes can have an impact on oocyte development, albeit the mechanisms still need to be investigated in depth.

## Conclusion

The study suggested that PFOA exposure was higher in the DOR group and PFOA may be a potential risk for DOR. However, there was no significant difference between high PFOA concentration and embryo quality in the DOR population, large-sample studies are required to confirm it.

## Supplementary Information

**Additional file 1:** **Supplementary table 1.** PFOA concentration in different groups.

## Data Availability

All data are included in this article and its supplementary information files.
